# Assessment of knowledge, attitudes, and practices regarding needlestick injuries among nurses in a public hospital in Malaysia

**DOI:** 10.3389/fpubh.2026.1774534

**Published:** 2026-02-25

**Authors:** Mohd Fahami Nur Azlina, Anchita Mottiakavandar, Tze Han Tang, Luo Yan Wong, Irliana Binti Ibrahim, Nafeeza Mohd Ismail

**Affiliations:** 1 Jabatan Farmakologi, Fakulti Perubatan, Universiti Kebangsaan Malaysia, Kuala Lumpur, Malaysia; 2 Department of Pharmacology, Faculty of Medicine, Universiti Teknologi MARA, Sungai Buloh Campus, Sungai Buloh, Selangor, Malaysia

**Keywords:** bloodborne pathogens, hospital, nurses, post-exposure prophylaxis, sharp objects

## Abstract

Needlestick injuries (NSIs) represent a significant occupational hazard among nurses, as they may result in serious consequences, including transmission of human immunodeficiency virus (HIV) and hepatitis B and C viruses. Therefore, this study primarily aimed to assess knowledge, attitudes, and practices (KAP) related to needlestick injury prevention among nurses at Hospital Canselor Tuanku Muhriz (HCTM) regarding NSIs. A cross-sectional study was conducted using a self-administered questionnaire distributed through universal sampling to 352 nurses at HCTM. Data collected included demographic characteristics and KAP scores related to NSIs. Among the respondents, 14.4% self-reported history of NSI during their professional duties, with 9.9% reporting at least one such event within their current department. No significant association was identified between a self-reported history of NSI and the nurses’ practice scores (*p* > 0.05). Furthermore, no significant correlations were identified between knowledge and attitude (r = −0.034, *p* > 0.05), knowledge and practice (r = 0.020, *p* > 0.05), or attitude and practice (r = 0.151, *p* > 0.05) among nurses at HCTM. Overall, the findings indicate that nurses demonstrated relatively moderate levels of knowledge, and relative high attitude, and practices related to NSIs. Despite these levels, self-reported needlestick injuries were still present among respondents. Within the limitations of this cross-sectional analysis, the findings suggest that individual KAP scores were not statistically correlated with NSI occurrence. System-level factors, including organizational conditions and institutional support, should be considered when developing strategies to strengthen needlestick injury prevention in healthcare settings.

## 1 Introduction

According to the Centers for Disease Control and Prevention (CDC), a needlestick injury (NSI) is defined as an accidental penetrating wound to the skin caused by a hollow-bore needle or any sharp object that is contaminated with another person’s blood or body fluids (1). In contrast, a sharp injury (SI) refers more broadly to penetrating skin injuries caused by sharp instruments occurring in the healthcare setting. Both needlestick injuries and other sharps injuries represent significant occupational health and safety concerns among healthcare workers (HCWs) worldwide, as they pose substantial risk for the transmission of blood-borne pathogens, including hepatitis B virus (HBV), hepatitis C virus (HCV), and human immunodeficiency virus (HIV), through cuts, puncture, or splashes in the workplace.

In addition to blood-borne infections, HCWs, particularly nurses, are at risk of acquiring approximately 20 other infections transmissible via needles, including tuberculosis, syphilis, and malaria (2). Globally, among an estimated 39.5 million HCWs, approximately 3 million experience NSIs annually (3). The occurance of NSIs varies across countries and is influenced by the level of economic development. In Malaysia, the reported occurrence was six NSI cases per 1,000 healthcare workers 2016 (4).

Within healthcare environments, sharp instruments such as needles and ampoules are routinely handled during daily clinical activities, posing a significant threat to the physical and psychophysical well-being of HCWs, particularly nurses. The estimated annual global risk of occupational exposure to blood-borne pathogens among HCWs ranges from 2.7–10.0% for HCV, 1.9–40% for HBV, and 0.2–0.44% for HIV (5). A study conducted in Qatar reported that operating theatres accounted for the highest proportion of NSIs (31.5%), followed by inpatient wards (21.6%), emergency departments (18%), intensive care units (8.1%), and other settings, including delivery rooms, laboratories, outpatient clinics, and ambulances (6).

Under the Occupational Safety and Health Act of 1994 (OSHA), sharp injuries are considered preventable. The Ministry of Health Malaysia (MOH) has identified NSI as a national indicator under the healthcare quality assurance programme, with a target of zero. However, studies have shown persistent unsafe practices among HCWs. For example, Bhargava, Mishra (7) reported that 37% of doctors, 19% of nurses, 31% of technicians, and 58% attendants believed that recapping or bending needles after use was acceptable, despite needle recapping being prohibited under OSHA blood-borne pathogen standards. Consequently, prompt exposure management, including the administration of post-exposure prophylaxis (PEP), crucial in reducing the risk of infection following accidental occupational exposure. Nevertheless, awareness of PEP remains suboptimal; one study reported that only 10% of HCWs were knowledgeable about PEP (8).

Recent evidence indicates that NSIs continues to occur in Malaysian healthcare settings. A study conducted in Malaysia reported that 23.6% of nurses indicated a self-reported history of needlestick injury in Selangor (4, 9). Similarly, an earlier study conducted at Hospital Canselor Tunku Muhriz, Universiti Kebangsaan Malaysia (HCTM), found that 34.9% of house officers experienced NSIs during the training period (10).

Given the significance of this occupational hazard and the limited number of studies focusing on nurses in Malaysian teaching hospitals, the present study was conducted to assess knowledge, attitudes, and practices related to needlestick injury prevention and to describe self-reported needlestick injury occurrence among nurses at a teaching hospital in Kuala Lumpur. The findings are expected to inform the development of targeted preventive strategies aimed at reducing the occurrence of such injuries. Therefore, the primary objective of this study was to assess knowledge, attitudes, and practices related to needlestick injury prevention among nurses at HCTM. A secondary objective was to describe self-reported needlestick injury occurrence within the preceding one-year period.

## 2 Methods

### 2.1 Study design and participants

A cross-sectional study was conducted in April 2023 among nurses at Hospital Canselor Tunku Muhriz (HCTM). A universal sampling approach was employed, whereby all eligible nurses were invited to participate. A total of 354 nurses (26.8%) who provided informed consent were included in the study. All participants were informed about the study design and objectives before their participation. Participants’ anonymity and confidentiality were strictly maintained throughout the study.

### 2.2 Data collection instrument and scoring

Data were collected using a validated, self-administered questionnaire designed to identify predictive factors associated with NSIs. The instrument collected both qualitative and quantitative data and comprised several subscales, including demographic characteristics, knowledge, attitudes, and practices related to NSIs. Each subscale consisted of five items.

For scoring, correct responses were awarded two points, while incorrect responses received zero points, resulting in a maximum score of 10 for each subscale. For analytical purposes, study-defined thresholds were applied to categorize KAP scores. Scores ranging from 0 to 4 were classified as low, 5–6 as moderate, and ≥7 as higher-level. These cut-offs were adapted from previous KAP studies (7, 11) to facilitate comparison across studies and do not represent validated safety standards or absence of occupational risk. These thresholds were used for analytical comparison with previous studies and do not imply absence of occupational risk. Knowledge, attitude, and practice (KAP) scores were calculated for each respondent and analyzed according to demographic and professional characteristics, including years of experience and educational background. The categorization thresholds were adapted from prior KAP studies and are intended to facilitate comparative analysis rather than to indicate complete safety or absence of occupational risk.

### 2.3 Statistical analysis

Data were analysed using IBM SPSS Statistics version 29.0. Descriptive statistics were presented as frequencies, percentages, means, standard deviations, and medians as appropriate. Bivariate parametric analyzed were conducted to determine associations between KAP scores and repondents’ professional and demographic characteristics, including years of experience, current department, and educational level. Statistical significance was set at *p* < 0.05.

### 2.4 Ethical approval

Ethical approval for this study was obtained UKM Research Ethics Committee (Approval code: JEP-2023-361). Permission to use the questionnaire was also obtained from the original developer. Participation in the study was voluntary, and informed consent was obtained from all respondents prior to data collection.

## 3 Result

### 3.1 Response rate and demographic characteristics

Out of an estimated 1,317 nurses employed at HCTM in 2022 a total of 352 nurses consented to participate in this study, resulting in a response rate of 26.8% (Table 1).

**Table 1:** Demographic characteristics of respondents (*N* = 352).

Variable	Category	*N* (%)
Gender	Male	23 (6.5)
	Female	329 (93.5)
Age (years)	21–20	48 (13.6)
	31–40	151 (42.9)
	41–50	143 (40.6)
	51–60	10 (2.8)
Highest educational qualification	Diploma	269 (76.4)
	Bachelor’s degree	72 (20.5)
	Master’s degree	3 (0.9)
	Others	8 (2.3)
Current department	Coronary Care Unit (CCU)	6 (1.7)
	Cardiac Rehabilitation Ward (CRW)	4 (1.1)
	Emergency Department (ED)	13 (3.7)
	Intensive Care Unit (ICU)	25 (7.1)
	Medical	55 (15.6)
	Obstetrics and Gynaecology (O&G)	59 (16.8)
	Orthopaedics	44 (12.5)
	Operating Theatre (OT)	29 (8.2)
	Outpatient Clinic	34 (9.7)
	Psychiatry	36 (10.2)
	Surgical	33 (9.4)
	Geriatric	14 (4.0)
Years of working experience	< 4 years	32 (9.1)
	5–10 years	58 (16.5)
	>11 years	262 (74.4)
Self-reported history of needlestick injury	Yes	51 (14.5)
	No	301 (85.5)
Number of self-reported NSIs in the past year	0	314 (89.2)
	1	30 (8.5)
	>1	8 (2.3)
Number of self-reported NSIs in current department	0	317 (90.1)
	1	22 (6.2)
	2	5 (1.4)
	3	5 (1.4)
	4	1 (0.3)
	>4	2 (0.6)

NSI, Needlestick injury; N, number.

The majority of participants were female (93.5%). Most nurses were aged between 31 and 40 years (42.9%), followed by those aged 41–50 years (40.6%). In terms of educational attainment, 76.4% held a diploma qualification, while 20.5% possessed a bachelor’s degree. Participants were distributed across 12 clinical departments at HCTM, with the highest proportion from the Obstetrics and Gynaecology (O&G) Department (16.8%).

In terms of professional experience, most participants reported more than 11 years of working experience (74.4%). A total of 14.5% reported a history of NSI, and 10.8% experienced at least one NSI in the past year. A total of 9.9% of participants reported at least one NSI within their current department.

### 3.2 Knowledge, attitudes, and practices of nurses toward needlestick injuries

Table 2 summarizes the knowledge, attitudes, and practices of nurses regarding NSI. Overall, nurses demonstrated moderate knowledge and relatively high attitudes and self-reported preventive practices. About knowledge, 67.9% of respondents correctly identified the appropriate immediate action following NSI, and almost all nurses (99.7%) were aware of the correct department for reporting NSI incidents. However, 88.6% incorrectly identified the relative risk of hepatitis B virus (HBV) transmission compared with human immunodeficiency virus (HIV), and 65.6% were unaware of the correct risk of transmission for HIV and HBV. In contrast, most respondents (85.8%) correctly recognized that the hepatitis C virus (HCV) can be transmitted via NSI.

**Table 2:** Distribution of correct responses and self-reported safety behaviors related to needlestick injury prevention (*n* = 352).

Item	Correct, *n* (%)	Incorrect, *n* (%)
Knowledge		
Correct immediate action after NSI	239 (67.9)	113 (32.1)
Higher risk of HBV transmission than HIV following NSI	40 (11.4)	312 (88.6)
Correct department for NSI reporting	351 (99.7)	1 (0.3)
HCV transmission possible via NSI	302 (85.8)	50 (14.2)
Correct HIV and HBV risk of transmission after NSI	121 (34.4)	231 (65.6)
Attitudes		
Needles should not be recapped or bent after use	337 (95.7)	15 (4.3)
Post-exposure prophylaxis is necessary	298 (84.7)	54 (15.3)
NSI incidents should be reported	352 (100.0)	0 (0.0)
Needles should be discarded immediately after use	348 (98.9)	4 (1.1)
Gloves provide protection against NSI	143 (40.6)	209 (59.4)
Practices		
Use of sharps container after phlebotomy	342 (97.2)	10 (2.8)
Vaccinated against HBV	280 (79.5)	72 (20.5)
No recapping of needles after use	338 (96.0)	14 (4.0)
Self-reporting of needlestick injury	346 (98.3)	6 (1.7)
Use of gloves during phlebotomy	342 (97.2)	10 (2.8)

HBV, Hepatitis B virus; HIV, Human immunodeficiency virus; HCV, Hepatitis C virus; NSI, Needlestick injury.

In terms of attitudes, 95.7% of nurses agreed that needles should not be recapped or bent after use, and 96.0% reported adhering to this recommended practice. A total of 84.7% of participants agreed that PEP is necessary in clinical practice. All nurses were aware that NSI incidents should be reported promptly; nevertheless, 1.7% admitted to not reporting such incidents. Additionally, 98.9% recognized that needles should be disposed of immediately after use. Although only 40.4% of nurses were aware that gloves can protect NSI, 97.2% reported wearing gloves for protection.

Regarding practices, 97.2% reported using sharps containers after phlebotomy procedures, 79.5% had been vaccinated against HBV, and 96.0% did not recap needles after use. Almost all respondents indicated that they would report NSI incidents (98.3%) and wear gloves during phlebotomy procedures (97.2%).

### 3.3 Association between KAP scores and demographic characteristics

The mean knowledge score was 6.11, indicating a moderate level of knowledge regarding NSI. As shown in Table 3, no statistically significant associations were observed between mean knowledge scores and demographic characteristics, including age, gender, educational level, years of experience, or history of NSI.

**Table 3:** Mean knowledge scores according to demographic characteristics (*n* = 352).

Demographic variables	Category	Mean ± SD	*p*-value
Age (years)	21–30	5.91 ± 1.37	0.580
	31–40	6.07 ± 1.68	
	41–50	5.87 ± 1.60	
	51–60	6.40 ± 1.58	
Gender	Male	5.91 ± 1.76	0.829
	Female	5.98 ± 1.58	
Education level	Diploma	6.01 ± 1.55	0.735
	Bachelor’s degree	5.83 ± 1.77	
	Master’s degree	6.66 ± 1.15	
	Others	6.00 ± 2.00	
Years of experience	<4 years	5.93 ± 1.64	0.406
	5–10 years	6.24 ± 1.41	
	>11 years	5.93 ± 1.64	
Self-reported history of NSI	No	5.96 ± 1.60	0.517
	Yes	6.11 ± 1.62	

SD, Standard deviations; NSI, Needlestick injury.

The mean attitude score among nurses was 8.22, reflecting a relatively high levels of attitude toward NSI. A statistically significant association was found between educational level and attitude score (*p* = 0.016) (Table 4). In contrast, no significant associations were found between attitude scores and age, gender, years of working experience, or history of NSI (*p* < 0.05).

**Table 4:** Mean attitude scores according to demographic characteristics (*n* = 352).

Demographic variables	Category	Mean ± SD	*p*- value
Age (years)	21–30	8.31 ± 0.689	0.339
	31–40	8.32 ± 0.638	
	41–50	8.22 ± 0.641	
	51–60	8.50 ± 0.527	
Gender	Male	8.28 ± 0.640	0.624
	Female	8.35 ± 0.714	
Education level	Diploma	8.26 ± 0.640	0.016*
	Bachelor’s degree	8.29 ± 0.638	
	Master’s degree	8.33 ± 0.577	
	Others	9.00 ± 0.535	
Years of experience	< 4 years	8.47 ± 0.879	0.234
	5–10 years	8.28 ± 0.686	
	> 11 years	8.26 ± 0.622	
Self-reported history of NSI	No	8.22 ± 0.642	0.413
	Yes	8.30 ± 0.645	

NSI, Needlestick injury. *A *p* < 0.05 is considered as significant value.

The mean practice score among nurses was 9.36, indicating a relatively high levels of practice related to NSI prevention. Statistically significant associations were observed between mean practice score and age (*p* = 0.001), gender (*p* = 0.044), and educational level (*p* = 0.028) (Table 5).

**Table 5:** Mean practice scores according to demographic characteristics (*n* = 352).

Demographic variables	Category	Mean ± SD	*p*- value
Age (years)	21–30	9.04 ± 1.368	0.001*
	31–40	9.54 ± 0.908	
	41–50	9.37 ± 1.243	
	51–60	8.20 ± 2.741	
Gender	Male	8.87 ± 1.180	0.044*
	Female	9.40 ± 1.216	
Education level	Diploma	9.31 ± 1.286	0.028*
	Bachelor’s degree	9.64 ± 0.844	
	Master’s degree	10.00 ± 0.000	
	Others	8.50 ± 1.414	
Years of experience	< 4 years	9.00 ± 1.437	0.173
	5–10 years	9.31 ± 1.096	
	> 11 years	9.42 ± 1.213	
Self-reported history of NSI	No	9.33 ± 1.239	0.194
	Yes	9.57 ± 1.082	

NSI, Needlestick injury. * A *p* < 0.05 is considered as significant value.

### 3.4 Correlation between knowledge, attitudes, and practices

Spearman’s correlation analysis showed no statistically significant correlations between knowledge and attitude, knowledge and practice, or attitude and practice (Table 6).

**Table 6:** Spearman’s correlation between knowledge, attitudes, and practices.

Variables	r	*P*-value
Knowledge-attitudes	−0.034	0.810
Knowledge-practice	0.020	0.702
Attitude-practice	0.151	0.291

Spearman’s rank correlation coefficient was used for analysis.

### 3.5 Association between knowledge, attitude, and practice levels and demographic characteristics among nurses with a history of needlestick injury

Table 7 presents a summary of the study’s findings on the levels of knowledge, attitudes, and practices among nurses with a history of NSI, as well as their associations with demographic characteristics. Among nurses with a history of NSI, knowledge levels were significantly associated with age, educational level, and years of work experience (*p* < 0.05), while no significant association was observed with gender. In contrast, attitude and practice levels showed no significant associations with any demographic variable (*p* > 0.05). These findings suggest that demographic factors influence knowledge acquisition related to NSI, but do not appear to translate into differences in attitudes or reported safety practices among nurses affected by NSI.

**Table 7:** Distribution of knowledge, attitudes, and self-reported practices among nurses with a history of needlestick injury.

Demographic variables	Knowledge			*p*-value	Attitudes			*p*-value	Practices			*p*-value
	Higher-level	Moderate	Lower level		Higher-level	Moderate	Lower level		Higher-level	Moderate	Lower level	
Gender												
Male	1	0	0	> 0.05	42	8	0	>0.05	49	0	1	>0.05
Gender	15	24	11		1	0	0		1	0	0	
Age (years)												
21–30	1	1	1	< 0.05*	3	0	0	>0.05	3	0	0	>0.05
31–40	9	7	7		19	4	0		23	0	0	
41–50	6	16	3		21	4	0		24	0	1	
51–60	0	0	0		0	0	0		0	0	0	
Education level												
Diploma	9	17	9	< 0.05*	28	6	0	>0.05	33	0	1	>0.05
Bachelor’s degree	7	5	2		12	2	0		14	0	0	
Master’s degree	0	2	0		2	0	0		2	0	0	
Others	0	0	0		1	0	0		1	0	0	
Years of experience												
<4	1	0	0	< 0.05*	1	0	0	>0.05	1	0	0	>0.05
5–10	0	3	2		5	0	0		5	0	0	
>11	15	21	9		37	8	0		44	0	1	

A *p*-value < 0.05 is considered as significant value.

## 4 Discussion

This study provides valuable insight into the knowledge, attitudes, and practices of nurses regarding NSI at HCTM. Of the total nursing workforce, a proportion consented to participate, resulting a modest response rate. Although this response rate may reflect time constraints or limited research engagement in research activities, the sample size was sufficient to provide meaningful overview of NSI-related issues within the hospital setting. Despite generally high levels of attitudes and practices, the continued occurrence of NSI highlights persistent occupational risks faced by nurses.

The demographic profile of respondents was largely reflective of the nursing workforce in Malaysia with women comprising the majority of participants. This gender distribution is consistent with national and international trends, where nursing remains a female-dominated professions. In term of educational background, most nurses held diploma-level qualifications, which may partly explain the moderate level of NSI-related knowledge observed. This finding contrasts with the study by Yazid et al. (11), who reported higher levels of NSI-related knowledge among nurses in another Malaysian public hospital. This discrepancy may be attributed to differences in hospital settings, exposure to training programmes, or participant characteristics. Similarly, Nawafleh et al. (12) reported poor knowledge of NSI-related topics among nursing students in Jordan, particularly regarding appropriate post-exposure management. In that study, students selected inappropriate first-aid measures, such as squeezing or applying pressure to the puncture site, rather than the recommended practice of washing the affected area under running water. Overall, variations in NSI-related knowledge across studies may be influenced by differences in educational curricula, clinical experience, and healthcare culture across countries (13).

Although most nurses demonstrated positive attitudes and appropriate safety practices, misconceptions regarding the protective role of gloves were evident. Specifically, only a minority of respondents recognised gloves as protective against NSI, which contrasts with findings by Sriram (14), where most healthcare workers were wearing gloves at the time of injury. This finding, therefore, highlights the need to strengthen education on the role of personal protective equipment in preventing NSI.

Furthermore, practice scores were significantly associated with age, gender, and educational level. Higher practice scores among nurses aged 31–40 years may reflect an optimal balance of clinical experience and physical capability. In contrast, lower scores among older nurses may be related to age-associated changes in dexterity and stamina, as previously reported by Mahmood and Hamzah (15). Supporting this observation, a study conducted in Ethiopia reported a higher occurrence of NSI among nurses with less than 5 years of experience, supporting the importance of knowledge in safe needle-handling practices (16). Conversely, nurses in the 51–60 years age group exhibited the lowest mean practice score, which may be attributed to age-related declines in physical stamina and manual dexterity (15).

In addition, gender-related differences in practice scores observed in this study align with findings by Saadeh et al. (17), suggesting that workplace safety behaviors may vary between male and female nurses. Notably, male nurses may encounter specific occupational barriers or be more exposed to risks associated with NSI. Moreover, nurses with higher educational attainment tended to demonstrate better mean practice scores, whereas those with lower educational levels showed comparatively lower scores. Overall, these results underscore the importance of implementing comprehensive NSI prevention programmes for nurses that incorporate not only training but also effective monitoring to ensure adherence to appropriate safety practices in the workplace.

Notably, the absence of significant correlations between knowledge, attitudes, and practices indicates that adequate knowledge alone does not necessarily translate into safer clinical practice. Although nurses may be aware of recommended guidelines for preventing NSI, the implementation of such knowledge in daily practice can be influenced by multiple factors, including workload, time constraints, availability of protective equipment, and institutional enforcement of safety policies (18). Emerging evidence supports this interpretation. Studies conducted in crisis-affected and high-stress healthcare settings demonstrate that psychological strain, reduced resilience, and organizational instability can undermine safe clinical practice even when knowledge and training are adequate. Aqtam et al. (19) reported that nursing students exposed to political violence exhibited lower adherence to safe clinical practices, which was associated with reduced psychological resilience and diminished perceived control within clinical settings. Similarly, studies conducted during the COVID-19 pandemic reported that increased occupational stress, moral distress, and organizational strain among intensive care nurses were associated with suboptimal safety behaviors despite prior training and professional experience (20). These findings are consistent with the results of the present study, in which no significant associations were observed between knowledge, attitudes, and practices. These findings underscore the importance of organizational and system-level interventions that move beyond education alone and incorporate continuous skills-based training and ongoing supervision to promote safe practices across healthcare settings.

Finally, the findings of this study can inform the planning of future interventions, not only at the study site but also in other hospital settings. Nevertheless, several limitations should be acknowledged. First, the response rate was modest, which may introduce selection bias. Nurses with greater awareness of occupational safety or prior experience with needlestick injuries may have been more likely to participate, potentially leading to an overestimation of knowledge and reported safety practices. Consequently, the findings should be interpreted cautiously and may not fully reflect the experiences of nurses with lower safety awareness or engagement. As such, the findings may not fully represent the broader nursing workforce at the study hospital or other healthcare settings. Second, data were self-reported and may be subject to recall and social desirability bias. Finally, the cross-sectional design limits the ability to infer temporal or causal relationships between knowledge, attitudes, practices, and injury occurrence.

## 5 Conclusion

This study found that nurses demonstrated moderate knowledge and relatively high attitudes and self-reported preventive practices related to needlestick injury prevention. Nevertheless, self-reported needlestick injuries were still observed among nurses at the study hospital, indicating that occupational exposure remains present despite reported awareness and compliance. These findings suggest that higher levels of knowledge and reported practice do not necessarily correspond to lower injury occurrence in a cross-sectional context. The results highlight the limitations of relying on individual-level training alone and underscore the importance of system-level approaches, including institutional safety culture, enforcement of safety policies, organizational support, and working conditions. Preventive strategies should therefore emphasize structural and organizational factors alongside continued education to support consistent implementation of safety practices across healthcare settings. Such programmes may enhance awareness of NSI hazards and ultimately reduce the risk of infection among healthcare workers and translating knowledge into sustained safe practice.

## Data Availability

The original contributions presented in the study are included in the article/supplementary material, further inquiries can be directed to the corresponding author.
